# A bayesian approach to model the underlying predictors of early recurrence and postoperative death in patients with colorectal Cancer

**DOI:** 10.1186/s12874-022-01746-y

**Published:** 2022-10-12

**Authors:** Leila Mahmoudi, Ramezan Fallah, Ghodratollah Roshanaei, Mohammad Asghari-Jafarabadi

**Affiliations:** 1 Department of Statistics and Epidemiology, School of Medicine, Zanjan University of Medical Sciences, Mahdavi Blvd, 4513956111 Zanjan, Iran; 2 Modeling of Non-communicable Diseases Research Canter, Department of Biostatistics, School of Public Health, Hamadan University of Medical Sciences, Hamadan, Iran; 3 Cabrini Research, Cabrini Health, Melbourne, VIC 3144 Australia; 4 School of Public Health and Preventative Medicine, Faculty of Medicine, Nursing and Health Sciences, Monash University, Melbourne, VIC 3800 Australia

**Keywords:** Bayesian, Semi-competing risks, Mortality, Early recurrence, Colorectal cancer

## Abstract

**Objective:**

This study aimed at utilizing a Bayesian approach semi-competing risks technique to model the underlying predictors of early recurrence and postoperative Death in patients with colorectal cancer (CRC).

**Methods:**

In this prospective cohort study, 284 patients with colorectal cancer, who underwent surgery, referred to Imam Khomeini clinic in Hamadan from 2001 to 2017. The primary outcomes were the probability of recurrence, the probability of Mortality without recurrence, and the probability of Mortality after recurrence. The patients ‘recurrence status was determined from patients’ records. The Bayesian survival modeling was carried out by semi-competing risks illness-death models, with accelerated failure time (AFT) approach, in R 4.1 software. The best model was chosen according to the lowest deviance information criterion (DIC) and highest logarithm of the pseudo marginal likelihood (LPML).

**Results:**

The log-normal model (DIC = 1633, LPML = -811), was the optimal model. The results showed that gender(Time Ratio = 0.764: 95% Confidence Interval = 0.456–0.855), age at diagnosis (0.764: 0.538–0.935 ), T_3_ stage (0601: 0.530–0.713), N_2_ stage (0.714: 0.577–0.935 ), tumor size (0.709: 0.610–0.929), grade of differentiation at poor (0.856: 0.733–0.988), and moderate (0.648: 0.503–0.955) levels, and the number of chemotherapies (1.583: 1.367–1.863) were significantly related to recurrence. Also, age at diagnosis (0.396: 0.313–0.532), metastasis to other sites (0.566: 0.490–0.835), T_3_ stage (0.363: 0.592 − 0.301), T_4_ stage (0.434: 0.347–0.545), grade of differentiation at moderate level (0.527: 0.387–0.674), tumor size (0.595: 0.500–0.679), and the number of chemotherapies (1.541: 1.332–2.243) were the significantly predicted the death. Also, age at diagnosis (0.659: 0.559–0.803), and the number of chemotherapies (2.029: 1.792–2.191) were significantly related to mortality after recurrence.

**Conclusion:**

According to specific results obtained from the optimal Bayesian log-normal model for terminal and non-terminal events, appropriate screening strategies and the earlier detection of CRC leads to substantial improvements in the survival of patients.

## Introduction

The third leading cause of cancer death is colorectal cancer, with a high level of burden [1]. Surveillance, Epidemiology, and End Results (SEER) 18 registry showed an incidence rate of up to 3.56 per 100,000 in gastrointestinal sites [2]] [[3]. The prevalence of colorectal cancer is higher in men than women [4]. Another study showed an increasing recent trends in colorectal cancer mortality [5]. Given that the burden of cancer is increasing, the goal is to reduce mortality from such non-communicable diseases by 2030. The burden of colorectal cancer can be reduced by intervening on modifying factors such as diet, lifestyle, and early detection of polyps using screening [6].

Considering that surgery as the initial treatment, the recurrence rate in the first 5 years after surgery is 12.8% for local recurrence and 25.6% for distant metastasis [7] [8]. In addition, 60–80% of recurrences of colorectal cancer appear in two years after surgery [5]. These patients have low survival if early recurrence occurs [6]. If recurrence and metastasis of the disease are diagnosed early, it may be possible to improve survival with curative surgery [7] [8]. By predicting recurrence and metastasis, appropriate treatment of patients with colorectal cancer can be prescribed after surgery [9]. The main goal of follow-up programs after colorectal cancer treatment is to increase survival.

Several studies have been carried out to study the risk factors of recurrence and survival in patients with colorectal cancer. The 3 and five year survival rates in patients without recurrence were 88.4% and 87.6%, respectively. Also, results showed that pT4 (HR: 4.06, 95%CI: 1.60-10.29, p = 0.003) was a risk factor for mortality [9]. Another study was conducted to recognize factors affecting recurrence in patients with colorectal cancer at a regional Australian hospital [10]. The study by Heinemann and Karl aimed to provide a brief overview of clinics, diagnosis, and management of some of the best colorectal cancer predispositions in this regard [11]. The incidence of colorectal cancer was lower in men than in women, so it was tried to improve the results of colorectal cancer in women by introducing new gender-specific methods [12]. In a five year cohort study, the effect of recurrence risk factors in patients with CRC after initial treatment revealed the effect of age, tumor location, lymphovascular invasion, and tumor stage on patient recurrence was significant [13]. Lymphovascular invasion, carcinoembryonic antigen (CEA), and prognostic factors, including metastatic and venous invasion, were identified as the risk factors for recurrence in colon and rectal cancers [14].

Depending on the study conditions and the characteristics of patients, various factors may affect the recurrence and the interval between recurrence and death, and studies have shown that there is no appropriate agreement for its determinants [5] [6] [9]. On the other hand, semi-competing risks refer to situations in which the main scientific interest in estimating and inferring concerning a non-terminal event (e.g., premature recurrence), the occurrence of which depends on a terminal event (e.g., death) [15]. Each of these must be appropriately considered so as not to cause bias in the results [16]. Few articles have considered them simultaneously, and most have been within the framework of the Cox model for the hazard function. Bayesian framework semi-competing risks modelling, wherein data may include left-truncation and, or interval-censoring, are very robust. Therefore, this study aimed to identify the predictors of recurrence, death and death after recurrence after curative surgery in patients with colorectal cancer, utilizing a semi-competing risk approach under the illness-death model.

## Methods

### Study design and setting

In this prospective cohort study, 284 patients with colorectal cancer, who underwent surgery, referred to Imam Khomeini clinic in Hamadan from 2001 to 2017.

### Predictors

All demographic and clinical/pathological information were extracted from patients’ records. These included demographic variables such as age at the time of diagnosis (years), gender (female:1; male:2), and Body Mass Index (BMI: kg/m^2^), and clinical/pathological variables such as metastasis to other sites (no:0; yes:1), cancer site (colon:1; rectum:2), surgery (no:0; yes:1), radiotherapy (no:0; yes:1), chemotherapy (no:0; yes:1), number of chemotherapy (0:no; 1:<6; 2:6+), morphology (0:no adeno; 1:adeno), grade (differentiation level) (1:well; 2:moderate; 3:poor), tumor size (1:<4; 2: >=4 < 7; 3:=>7), disease stage(1:B; 2:C;3:D), PT-stage(1:T2; 2:T3; 3:T4; 4:Tx), and PN-stage(1:N2; 2:N3; 3:N4; 4:Nx).

### Main outcome variables

Patients’ recurrence status was determined from patients’ records. The time to recurrence of patients, the non-terminal event, was computed from the date of surgery to local or distant recurrence in months (totally considered to experience the non-terminal event), and individuals who did not have recurrence or death until the end of the study were considered as censors. The death of the patients, the terminal event, was computed from the surgery date to their death according to the researchers’ telephone follow-up. Also, regarding the follow-up and the need to be in contact after the illness, the exact address and two contact numbers were received and recorded in the patient’s file.

### Statistical analyses

Data were explained as mean (SD), median (min-max) for the normal and non-normal numeric variables, respectively, and frequency (percent) for categorical variables. The occurrence rate of the non-terminal event (recurrence) and the terminal event (death) was computed per 1000 persons. Log-rank tests were carried out to compare the survival rates across age at diagnosis, gender, BMI, metastasis to other sites, cancer site, surgery, radiotherapy, and chemotherapy, number of chemotherapy, morphology, grade of differentiation, tumor size, disease stage, PT-stage, and PN-stage. Also, the adjusted survival curves were plotted for significant variables in the multivariable analysis. These parts of the studies were conducted using Stata17 software (StataCorp, College Station, Texas, USA). To assess the relationship of above-mentioned variables with outcomes including the probability of the non-terminal event, the probability of the terminal event, and the conditional probability of the terminal event after non-terminal event, semi-competing risks analysis was utilized under the illness-death model with AFT approach. These outcomes were specified by three hazard functions in the Bayesian illness-death models. Accordingly, a Gibbs random sampling algorithm was used to generate samples from the complete posterior distribution. Deviance information criterion (DIC) and Logarithm of the pseudo marginal likelihood (LPML) were considered to compare the models.

### Accelerated failure time models for independent semi-competing risks data

The AFT assumption can be used to compare survival times. One of the AFT model assumptions is that the effect of covariates on survival time is multiplicative [17]. The following AFT model was considered for the data analysis

1 $${\rm{log(}}{{\rm{T}}_{{\rm{i1}}}}{\rm{) = X}}_{{\rm{i1}}}^{\rm{T}}{{\rm{\beta }}_{\rm{1}}}{\rm{ + }}{{\rm{\gamma }}_{\rm{i}}}{\rm{ + }}{{\rm{\varepsilon }}_{{\rm{i1}}}}{\rm{,}}{{\rm{T}}_{{\rm{i1}}}}{\rm{ > 0}}$$

2 $${\rm{log(}}{{\rm{T}}_{{\rm{i2}}}}{\rm{) = X}}_{{\rm{i2}}}^{\rm{T}}{{\rm{\beta }}_{\rm{2}}}{\rm{ + }}{{\rm{\gamma }}_{\rm{i}}}{\rm{ + }}{{\rm{\varepsilon }}_{{\rm{i2}}}}{\rm{,}}{{\rm{T}}_{{\rm{i2}}}}{\rm{ > 0}}$$

3 $${\rm{log(}}{{\rm{T}}_{{\rm{i2}}}}{\rm{ - }}{{\rm{T}}_{{\rm{i1}}}}{\rm{) = X}}_{{\rm{i3}}}^{\rm{T}}{{\rm{\beta }}_{\rm{3}}}{\rm{ + }}{{\rm{\gamma }}_{\rm{i}}}{\rm{ + }}{{\rm{\varepsilon }}_{{\rm{i3}}}}{\rm{, }}{{\rm{T}}_{{\rm{i1}}}}{\rm{ > }}{{\rm{T}}_{{\rm{i2}}}}$$

Ti1 and Ti2 were considered as times to the non-terminal and terminal events, respectively. In each of the equations above, let x_ig_ be a vector of transition-specific covariates, let βg denote a vector of transition-specific regression parameters, and it is assumed ε_ig_ is a transition-specific random variable, g = 1, 2, 3. Also, in each of expressions (1)–(3), γi is a study subject-specific frailty that instills positive dependence between the non-terminal and terminal events and It is assumed that γi is adopted from a normal distribution with mean of zero and variance of θ. In addition, it is considered the variance component θ adopted a conjugated inverse gamma distribution, which is defined by IG (a(θ), b(θ)). For regression parameters, β_g_ is adopted non-informative flat prior on the real line parametric modeling, which was built on the log-normal distribution, and take the ε_ig_ follows a normal (µ_g_, σ^2^_g_) distribution. For µ_g_, was assumed non-informative flat priors on the real line and for σ^2^_g_, independent inverse Gamma distributions, denoted by IG (a_g_^(σ)^, b_g_^(σ)^).

To enrich the study, also was used a semi-parametric framework. In many cases, due to the unrealistic features of some common models, including the thin tail of the normal distribution, compared to the observed data distribution, the results of parametric models are not satisfactory, therefore, semi-parametric models can enrich the study.

So for each ε_ig_ was adopted an independent non-parametric Dirichlet process mixtures (DPM) of M_g_ normal distributions with mean µ_gr_ and variance σ^2^_gr_, r∈ {1… M_g_ }.

Bayesian models were compared with two effective measures, DIC and LPML for recognizing the true model. The smaller DIC values indicate that the model has a better fit for the data [18]. The larger LPML values also indicate that the model has a good fit for the data [19].

This part of the analyses were carried out using R 4.1 software utilizing a SemiCompRisks package [20]. The significance level was set at 0.05.

## Results

### Patients profile

Out of 284 patients with resected CRC, 150 (52.8%) were mal. A total of 121 (42.6%) patients died, and 131 (46.1%) patients had a recurrence, of which 105 (80.2%) patients died by the end of the study. In addition, there were 16 (10.5%) patients who experienced death without experiencing the recurrence. The recurrence rate was about 46% in the colon, and rectum cancer sites. The mean age at diagnosis was 55.6 (SD 13.1) years, with an age range of 21–84 years. In addition, the mean age at diagnosis in patients with and without recurrence was 56.7 (SD 13.4) and 54.7 (SD 12.8) years, respectively. The median survival of patients was 61.0 (95% Confidence Interval (CI): 42.2–79.8) months. Besides, median survival was 47.0 (95% CI: 21.0–73.0) months for patients with recurrence. The total percentage of recurrences by the end of the first, second, third, fourth, and fifth years were 64.1%, 82.4%, 89.3%, 93.9%, and 96.2%, respectively. Only 3.8% of recurrences occurred after five years and the median recurrence time in patients with and without recurrence was 7 and 46 months, respectively. Moreover, the 1-, 3-, 5- and 10-year survival probabilities were 86.9%, 62.1%, 50.4%, and 42.3%, respectively, for the terminal event, and the 1-, 3-, 5- and 10-year survival probabilities were 67.4%, 51.9%, 45.3%, and 40.3%, respectively for the non-terminal event. The mean and median time distance between non-terminal and terminal events was 26.2 (95% CI: 19.1–33.2) and 10.0 (95% CI: 7.8–12.2) months, respectively. After disease recurrence, 1-, 3-, 5- and 10-year survival probabilities were 67.4%, 51.9%, 45.3%, and 40.3%, respectively.

Also, among patients who had a recurrence by the end of the study, 110 (84%) had metastases to other sites, 12 (9.2%) did not attend chemotherapy sessions, and 76 (58%) attended more than six sessions. Seven (5.3%) were in stage T2 and 92 (70.2%) were in stage T3, 11 (8.4%) were in stage Nx, and 55 (42%) were in stage N0. Among patients who had died by the end of the study, 94 (77.7%) had metastases to other sites, 12 (9.9%) had not attended chemotherapy sessions, and 61 (50.4%) had attended more than six sessions, 7 (5.8%) were in stage T2, and 85 (70.3%) were in stage T3, 10 (8.3%) were in stage Nx, and 45 (37.2%) were in stage N1.

### Result of log-rank tests

According to the results of log-rank tests, age at diagnosis (years) (p = 0.001), metastasis to other sites (p = < 0.001), number of chemotherapies (p = 0.041), disease stage (p < 0.001), PT-stage (p < 0.001) and PN-stage (p < 0.001) were significant in both non-terminal and terminal events. In recurrence and dath outcomes, significantly higher outcome rates were observed among higher age categories, with substantially higher rates in age > 70. The rate of recurrences and death were 38.22, and 26.38, respectively. Also, those patients who had metastasis to other sites had much higher rates of both outcomes. The rate of recurrences and death were, 79.58, and 29.46, respectively. In addition, < 6 number of chemotherapies were associated with higher events than patients who had not had any chemotherapy. The rate of recurrences and death were, 17.40, and 16.38, respectively; however, the rates decreased when coming into 6 + chemotherapies. Non-terminal and terminal event rates raised significantly as the disease stage, PT-stage, and PN-stage levels increased a P < 0.05). Furthermore, comparing the occurrence rate in non-terminal and terminal events, it is evident that the occurrence rate is much higher in the recurrence than in the death outcome.

### Model comparison

For Bayesian Independent AFT model with log-normal baseline survival distribution, we observed a DIC = 1633 and a LPML = -811. As well as, for Bayesian Independent AFT model with DPM baseline survival distribution we observed a DIC = 1759 and a LPML =-816. According to DIC and LPML, the Bayesian Independent AFT model with log-normal baseline survival distribution was the best model, accordingly the results of this optimal model were followed.

### Result of bayesian AFT log-normal model

According to the results, the ratio of recurrence survival time was lower in men than in women (Time Ratio (TR) = 0.764: 95% CI 0.456–0.855). Age at diagnosis was associated with a lower survival time in all recurrence (0.764: 0.538–0.935), death without recurrence (0.396: 0.313–0.532) for, and death after recurrence (0.659: 0.559–0.803). Metastasis to other sites was associated with a lower time ratio of death without recurrence (0.566: 0.490–0.835). The number of chemotherapy sessions was associated with a higher survival time for all three recurrence (1.583: 1.367–1.863), death without recurrence (1.541: 1.332–2.243), and death after recurrence (2.029: 1.792–2.191). Grade of differentiation at moderate level was associated with a lower survival time for recurrence (0.648: 0.503–0.955) and death without recurrence (0.527: 0.387–0.674), however, at a weak level of differentiation, it was associated with a lower time ratio of recurrence (0.856: 0.733–0.988). Tumor size was linked with a lower time ratio for recurrence (0.709: 0.610–0.929), and for death without recurrence (0.595: 0.500–0.679). PT Stage at T3 was associated with a lower time ratio for recurrence (0.601: 0.530–0.713), and for death without recurrence (0.363: 0.592 − 0.301). T4 stage was associated with a lower time ratio for death without recurrence (0.434: 0.347–0.545). PN stage at N1 increased the time ratio for death without recurrence (1.974: 1.728–2.122) and N2 level also decreased the time ratio for recurrence (0.714: 0.577–0.935), (Table 1).

**Table 1:** Predictors of non-terminal and terminal events utilizing Bayesian Independent AFT model with log-Normal baseline survival distribution

		Recurrence		Death without recurrence		Death after recurrence	
		TR	95% CI	TR	95% CI	TR	95% CI
Age at Diagnosis (years)	Trend effect	0.764	0.538–0.935*	0.396	0.313–0.532 *	0.659	0.559–0803 *
Gender	Male	0.596	0.456–0.855 *	0.666	0.363–1.074	0.951	0.697–1.203
Metastasis to other sites	Yes	0.735	0.589–1.022	0.566	0.490–0.835*	0.821	0.594–1.052
Number of chemotherapies	Trend effect	1.583	1.367–1.863 *	1.541	1.332–2.243 *	2.029	1.792–2.191 *
Grade (differentiation level)	Well	Referent	---	---	---	---	---
	Moderate	0.648	0.503–0.955 *	0.527	0.387–0.674 *	1.053	0.839–1.277
	Poor	0.856	0.733–0.988 *	0.558	0.380–1.030	1.310	0.875–1.174
Tumor size	Trend effect	0.709	0.610–0.929 *	0.595	0.500–0.679 *	0.893	0.781–1.010
PT-Stage	T2	Referent	---	---	---	---	---
	T3	0.601	0.530–0.713 *	0.363	0.592 − 0.301 *	0.983	0.746–1.414
	T4	0.962	0.872–1.319	0.434	0.347–0.545*	0.853	0.509–1.402
	TX	NC	NC	NC	NC	NC	NC
PN-Stage	N0	Referent	---	---	---	---	---
	N1	1.272	0.967–1.586	1.947	1.728–2.122	0.760	0.576–1.468
	N2	0.714	0.577–0.935 *	1.302	1.000–1.620	0.802	0.592 − 0.086
	NX	NC	NC	NC	NC	NC	NC

NC: not computable; CI: Credibility interval; TR: Time Ratio

Deviance information criterion (DIC = 1633), Logarithm of the pseudo marginal likelihood (LPML= -811)

The frailty component was significant in the multivariable model (Variance of frailties: 0.245, 95% CI (0.209–0.281))

Trend effect: The model considered the trend effect for ordinal categorical variables. The variables BMI category, cancer site, surgery, radiotherapy, chemotherapy, morphology, disease stage could not be entered in the model in the multivariable model (All P > 0.05)

*: P < 0.05

### Result of bayesian AFT DPM model

According to the results, the time ratio of recurrence (0.835) was lower in men than in women. Age at diagnosis was associated with a decrease in the time ratio for recurrence (0.956). Metastasis to other sites was associated with an increase in the time ratio of recurrence (1.063), and a decrease in the time ratio for recurrence after death (0.946). The number of chemotherapy sessions significantly increased the time ratio of death after recurrence (1.045), (Table 2).

**Table 2:** Predictors of non-terminal and terminal events utilizing Bayesian Independent AFT model with DPM baseline survival distribution

		Recurrence		Death without recurrence		Death after recurrence	
		TR	95% CI	TR	95% CI	TR	95% CI
Age at Diagnosis (years)	Trend effect	0.956	0.942–0.970 *	0.996	0.906–1.135	0.999	0.961–1.037
Gender	Male	0.835	0.624–0.972 *	1.006	0.945–1.177	0.977	0.977–1.129
Metastasis to other sites	Yes	1.063	1.063–1.086 *	1.071	0.925–1.106	0.946	0.944–0.969*
Number of chemotherapies	Trend effect	1.069	0.989–1.069	1.019	0.909–1.147	1.045	1.045–1.045
Grade (differentiation level)	Well	Referent	---	---	---	---	---
	Moderate	0.974	0.877–1.062	1.059	0.950–1.078	0.996	0.947–1.001
	Poor	0.944	0.721–0.961 *	1.067	0.852–1.225	0.966	0.834–0.975*
Tumor size	Trend effect	0.890	0.855–0.960 *	1.002	0.972–1.038	1.039	1.000- 1.206*
PT-Stage	T2	Referent	---	---	---	---	---
	T3	1.048	0.927–1.050	1.018	0.994–1.083	1.066	0.975–1.233
	T4	1.006	0.914–1.068	1.095	0.978–1.095	1.077	0.992–1.162
	TX	NC	NC	NC	NC	NC	NC
PN-Stage	N0	Referent	---	---	---	---	---
	N1	1.033	1.033–1.077*	1.147	1.019–1.196 *	1.057	1.019–1.057
	N2	1.048	0.939–1.252	1.075	1.009–1.251 *	0.874	0.835–0.877
	NX	NC	NC	NC	NC	NC	NC

NC: not computable; CI: Credibility interval; TR: Time Ratio

Deviance information criterion (DIC = 1759), Logarithm of the pseudo marginal likelihood (LPML= -816)

The frailty component was significant in the multivariable model (Variance of frailties: 0.847, 95% CI (0.75 − 0.018))

Trend effect: The model considered the trend effect for ordinal categorical variables. The variables BMI category, cancer site, surgery, radiotherapy, chemotherapy, morphology, disease stage could not be entered in the model in the multivariable model (All P > 0.05)

*: P < 0.05

## Discussion

This study aimed to utilize the Bayesian framework of semi-competing risks to model the effect of background and clinical characteristics on recurrence and postoperative death in patients with CRC. Therefore, the effect of these variables on the non-terminal event (recurrence), the probability of the terminal event (death without recurrence), and the conditional probability of the terminal event on the non-terminal event (death after recurrence). The results of this study demonstrate that, the Bayesian AFT log-normal model was the best model, consistent with DIC and LPML. Accordingly, the results showed that gender, age at diagnosis, T-stage, N-stage, tumor size, grade of differentiation, and number of chemotherapies were significantly related to the recurrence outcome. Also, age at diagnosis, metastasis to other sites, T-stage, grade of differentiation, tumor size, and the number of chemotherapies significantly predicted the death without recurrence. In addition, age at diagnosis, and the number of chemotherapies were significantly related to death after recurrence.

The illness-death model is utilized because of its association with common methods for survival analysis and also, its software is available although the hazard ratio, a commonly used measure of association in survival analysis. This is by no means the only measure that researchers may choose to calculate and report the outcomes. The AFT model has a perceptive physical explanation, and easily model the logarithm of the survival time over the explanatory variables [21]. There was a strong association between the terminal and non-terminal events in the current study, therefore, the simple utilization of a univariate survival model for the non-terminal event, would lead to an overestimation of the terminal event rates, because the analysis considers the terminal event as an independent censoring mechanism [22]. Utilizing semi-competing risk analysis, the terminal event is regarded as competing event, and the dependence between the two events is assumed to be part of the model specifications.

The Bayesian approach is a scientific and practical, and an alternative to the frequent approaches, and are simply possible due to computational advances and available software. Considering the analysis of semi-competing risks data, the proposed AFT illness-death model supply as a beneficial complement to the more traditional hazard-based approach [23][24]. In this study, a Bayesian framework was applied that allows to simultaneously address three important scientific goals in the semi-competing risk data settings: estimating regression parameters, describing the within-subject dependence between two event times, and predicting outcomes. Therefore, AFT models with frailty were fitted with log-normal parametric and DPM non-parametric baseline hazards functions.

Grzenda used a similar model to analyze the duration of the first job among young people. For this purpose, four Weibull, Gamma, Log-normal, and Log-Logistic models with the Bayesian approach were proposed. Based on the comparison of the models using the DIC index, the gamma model was a good fit for the data [25]. Lee outlined a new Bayesian framework for an AFT illness-death model, wherein DIC and LMPL indices were used to compare the models [26]. Ganjali conducted a study to evaluate the duration of unemployment in conditions where the proportional hazard assumption was not assumed. For this purpose Bayesian log-logistic, log-normal, and Weibull AFT models were used [27]. Marcus Abiso Arango utilized three common Bayesian joint models with AFT Weibull, log-normal, and log-logistics probability distributions, and they decided on Bayesian logistics model as the final model utilizing DIC, AIC, and BIC indices [28].

We utilized a non-informative prior distribution such as Jeffrey’s prior, because it gives inferential results similar to those of the best frequentist methods [29]. In Bayesian analysis, a balance is always seek for between prior information and information from data; in the one hand, the prior information should not overwhelm the evidence from the data, in the other hand, a strong enough prior is required to support weak evidence that usually comes from insufficient data. This, sometimes is decided after performing sensitivity analysis to check the dependence of the results on the choice of a prior, a controversial issues associated with Bayesian analysis. So, choosing non-informative priors can be a great solution to achieve this balance, as well, by assigning equal probabilities to all possible states of the parameter space, can rectify the subjectivity problem. Another positive point about the Bayesian analysis and considering a non-informative prior is that even if the prior is improper, the corresponding posterior distribution may still be proper [30].

Recurrence affects survival and death in the first five years after recurrence in patients with curative resection, as reported in some studies [31][32][33]. In some studies, the 5-year cumulative recurrence rates were 4.9%, 11.0%, and 23.5% for stage I, stage II, and stage III tumors, respectively [8]. In patients with colon cancer, local recurrence was less than in patients with rectal cancer [34].

In this study, the postoperative survival rate was decreased in older ages. In the line of this study, Baghestani showed that age at diagnosis was significantly related to a patient’s survival time [35], as well, some studies reported similar results [36][37]. Also, in other studies, age was significantly associated with local and distance recurrence [38][39][40]. Also, age was significantly associated with the survival of patients with colorectal cancer. In these studies, it has been reported that higher age was associated with a decrease in patient survival [41][42][43]. However, in some other studies, no significant association was reported [44][45][46][47]. In addition, several studies have shown a significant association between age and 5-year survival [48][40]. For that reason, early screening in adults to diagnose cases can increase the survival time ratio in patients with colorectal cancer.

The findings of several studies are in the line with the results of the current study, wherein the ratio of survival time was lower in men than women for recurrence outcome. Although in some studies, results have shown that sex was not significantly associated with survival time [49][36], in one study showed that men had lower survival than women [50], and in another study, 5-year survival in the second step was higher in women than men [40]. So appropriate screening strategies should be considered.

Metastasis to other sites was another factor that showed a significant association with non-terminal and terminal events. Other studies showed similar results [38][39]. The rate of grade I tumors was significantly upper in the group that had late metastasis (35.1% vs. 64.9%, P = 0.001)[51]. In this study metastasis to other sites was associated with decreased survival time of Death without experiencing recurrence. Another study showed that the liver and lung were the first and second well-known sites of recurrence, respectively [34]. As a result, patients should be under intensive care in this regard.

According to the current study, grade of differentiation and tumor size were associated with a decrease in recurrence survival time. Moderate differentiation grade and tumor size was associated with decreased time ratio for death without recurrence. in some studies, patients with stage III tumors had low recurrence rates [52][53].

As a complementary treatment after surgery, the number of chemotherapies was significantly related to greater survival of non-terminal and terminal events and non-terminal event condition of the terminal event. Several studies have reported that postoperative adjuvant therapy with fluorouracil and levamisole, as standard adjuvant chemotherapy, reduces mortality in patients with colorectal cancer [54][55], as well as, chemotherapy effectively reduced the recurrence [56][57]. Therefore, chemotherapy can be suggested to decline the hazard of recurrence and death.

In the present study, PT stages, and T-stages were associated with decreased time ratio for death without recurrence, and PN-stage, and PT-stages were associated with a decrease in recurrence survival time. It has been shown that T3 to T4 were significantly and effectively associated with stage in patients CRC [58], as well, PT-stage and PN-stage were significantly related to early recurrence [59]. Also it was reported that the mortality rate was higher in patients with higher stages of colon cancer [60][61]. According to a study, in the higher stages of CRC, the rate of local recurrence and metastasis has been shown to be higher [62]. PN-Stage hase been shown to be effective on recurrence [63]. Therefore, cancer extent in the body should be determined and appropriate treatment should be assigned according to the stage of cancer.

### Limitation of the study

As the first limitation, there was a difficulty in fitting Bayesian models, which was minimized by using appropriate approaches in modeling, selecting the appropriate initial values in the models, and selecting the appropriate amount of memory for the systems running the program. In particular, more cases are needed to achieve higher statistical precision. Another limitation of the present study was its generalizability, because the participants in this study were specific in terms of environmental, cultural, social, and geographical conditions, so the results of this study should be interpreted with cautions when would be generalize to other individuals and communities. There are some restricting assumptions in utilizing the proposed models in the current study, and only linear effects of the predictors were considered. Accordingly, machine learning methods such as neural networks, classification algorithms, and regression trees automatically consider linear and nonlinear interaction relationships and possibly provide more accurate results. For our upcoming project, we intend to follow machine learning methods.

## Conclusion

This study demonstrated, according to the Bayesian AFT log-normal model, as the best model, that gender, older age, higher pathological, higher T/N stage, and fewer chemotherapy sessions were significantly related to the lower survival time ratio of patients with CRC. According to specific results obtained for terminal and non-terminal events, appropriate screening strategies and the earlier detection of CRC may lead to substantial improvements in the survival of patients.

## Data Availability

The data that support the findings of this study are available from MAJ, but restrictions are applied to the availability of these data, which were used under license for the current study, and are not publicly available. Data are, however, available from the authors upon reasonable request by MAJ.
